# Multi-Scale Assessments of Cardiac Electrophysiology Reveal Regional Heterogeneity in Health and Disease

**DOI:** 10.3390/jcdd5010016

**Published:** 2018-03-08

**Authors:** Catherine E. Lipovsky, Brittany D. Brumback, Aditi Khandekar, Stacey L. Rentschler

**Affiliations:** 1 Department of Medicine, Cardiovascular Division, Washington University School of Medicine, Campus Box 8103, 660 S Euclid Ave, St. Louis, MO 63110, USA; clipovsky@wustl.edu (C.E.L.); bbrumback@wustl.edu (B.D.B.); aditiakhandekar@gmail.com (A.K.); 2 Department of Developmental Biology, Washington University School of Medicine, St. Louis, MO 63110, USA; 3 Department of Biomedical Engineering, Washington University, St. Louis, MO 63130, USA

**Keywords:** cardiac development, electrophysiology, optical mapping, ECGI

## Abstract

The left and right ventricles of the four-chambered heart have distinct developmental origins and functions. Chamber-specific developmental programming underlies the differential gene expression of ion channel subunits regulating cardiac electrophysiology that persists into adulthood. Here, we discuss regional specific electrical responses to genetic mutations and cardiac stressors, their clinical correlations, and describe many of the multi-scale techniques commonly used to analyze electrophysiological regional heterogeneity.

## 1. Introduction

The evolution of species from the sea onto land required the ability to oxygenate blood in the lungs. Coincident with the need for a pulmonic circulation, the embryo developed a way of recruiting additional cells into the heart to form the second ventricle. While the left ventricle (LV) is derived from cells within the first heart field, the cells that ultimately form the mature right ventricle (RV) derive from a distinct progenitor population defined as the second heart field. It is well known that LV and RV cardiomyocytes have distinct transcriptional profiles during development, and that their transcriptional profiles become more similar in the adult heart [1]. However, given that their progenitors are subjected to different signaling pathways throughout development, LV and RV transcriptional differences are often accentuated in response to genetic mutations or stress [2]. In this commentary, we will discuss how developmental programming can affect regional electrophysiology (EP) and arrhythmia predisposition, and we will delineate some of the techniques used to study regional heterogeneity within the heart.

## 2. Developmental Signals Regulating LV-RV Cardiomyocyte Programming

First and second heart field patterning is controlled by distinct transcriptional networks, with pathways, such as Hand1, predominantly active in the first heart field, while Hand2 and Wnt signaling play important functions during second heart field development [3,4,5]. Changes in chromatin accessibility throughout development and aging may, at least in part, explain why the timing of transcription factor expression is crucial. For example, activation of a signaling pathway may program a distinct cellular subtype during embryogenesis, while reactivation of the same pathway in the adult may result in only a partial phenotypic conversion. Activation of either neuregulin-1 or Notch is sufficient to convert embryonic ventricular myocytes into Purkinje cells, while in the adult, there is a more limited transcriptional response and only a subset of Purkinje-enriched ionic currents are modulated [6,7,8,9].

In addition to the importance of timing, regional developmental programming may result in distinct chamber-specific electrophysiological responses. To highlight one example, we performed single cell voltage clamp experiments on dissected left versus right ventricular tissue in Notch activated hearts and observed different electrical responses. While activation of Notch signaling prolongs the action potential duration (APD) and downregulates voltage-gated potassium currents in the LV, no change in APD or K^+^ current density was observed in the RV [8]. These electrical changes are mediated, at least in part, through altered transcription and epigenetic changes to the promoters of subunits encoding the ion channels. Coincident with the observed chamber-specific differences in ion channel subunit expression, activation of Notch upregulates the transcriptional repressor *Hairy-related transcription factor 2* (*Hrt2*, also known as *Hey2*) in the LV as expected, given that *Hey2* is a direct Notch target. In contrast, unexpectedly, Notch activation in the RV downregulates *Hey2* expression [8]. Our initial gene expression analysis conducted on pooled ventricular tissue from Notch-activated mice did not reveal changes in *Hey2* expression due to discordant chamber effects, and it was only after separating LV from RV that these chamber-specific effects were observed. This example underscores the idea that separately interrogating distinctly programmed regions, such as the LV and RV, may result in different insights. Interestingly, genome-wide association studies have implicated variants near several ion channels, as well as the 6q22.31 locus near *HEY2* with Brugada syndrome (BrS) [10]. BrS is characterized by ST-segment elevation and a propensity for sudden death due to RV-predominant arrhythmias. Therefore, a more precise understanding of how Notch and *Hey*2 regulate chamber-specific ion channel gene expression may yield important translational insights.

## 3. Diseases Associated with a Ventricular Chamber Predilection

It is well known that the LV and RV have distinct geometries and mechanical properties suited for either pumping blood against high systemic pressures to the body versus against lower pressures to the lungs. This concept is highlighted in a rare congenital cardiac malformation, congenitally corrected transposition of the great arteries (ccTGA), where both the atrioventricular and ventriculoarterial connections are discordant [11]. In this anomaly, the right atrium (RA) connects to the morphologic LV and pulmonary artery, and the left atrium connects to the morphologic RV and aorta [12]. While this structural malformation is compatible with survival into adulthood, the morphologic RV functions as the systemic pumping chamber. Given the inherent differences in morphologic LV and RV function, over time, systemic pressures in the morphologic RV lead to hypertrophy and subsequently to failure of the systemic ventricle [13].

In addition to properties that confer mechanical differences, unique transcriptional signatures of ion channel subunits confer distinct electrical functions to the LV and RV. This concept has emerged clinically with respect to genetic mutations specifically affecting one ventricle of the heart, despite all ventricular myocytes possessing the same genome. One disease that highlights differences between LV and RV cardiomyocytes is arrhythmogenic cardiomyopathy (ACM), a heritable condition predisposing to ventricular arrhythmias and sudden cardiac death. ACM was predominantly considered an RV-dominant disease, otherwise known as Arrhythmogenic Right Ventricular Cardiomyopathy (ARVC), until about a decade ago. Two more subtypes of ACM, biventricular ACM and LV-dominant ACM, have been identified and studied in the last few years, however, ARVC is still considered the most dominant subtype [14]. ARVC is characterized by fibro-fatty infiltrations, RV dilation, and arrhythmias originating from the RV [15]. Mutations in genes encoding desmosomal proteins are attributed as the genetic cause of ARVC, and recent studies have provided insight into the mechanism underlying how these genetic mutations give rise to ARVC [16]. Although much is still not understood about why this heritable disease tends to affect RV over LV, it underscores that there are distinct identities and signature roles for LV and RV cardiomyocytes in electrophysiologic homeostasis [16,17]. The final section of this review will describe various EP techniques that can be used to investigate cardiac electrical properties from single cells to the whole heart in both health and in diseases, such as ARVC.

## 4. Techniques to Study Regional Electrophysiological Differences

### 4.1. Multi-Scale Assessment of Cardiac Electrical Properties

Cardiomyocytes are excitable cells due to the movement of ions across the cell membrane. This ionic movement causes changes in membrane voltage, resulting in the cardiac action potential (AP). Cardiomyocytes are electrically coupled to each other through gap junctions, which allow the AP of a single cell to electrically stimulate a neighboring cell and ultimately to propagate throughout the cardiac tissue. Distinct regional expression of ion channels leads to the LV AP waveform morphology being distinguishable from that of the RV, and even the AP morphologies within the same chamber differ depending on location within the chamber wall (i.e., subepicardium vs. midmyocardium vs. subendocardium). These baseline differences in regional cardiac EP confer a unique susceptibility to arrhythmia predisposition in diseased states when ion channel function is affected.

Arrhythmias develop when electrical impulse activation and/or propagation are disrupted. For instance, atrial fibrillation is an arrhythmia in which ectopic electrical foci or “triggers” originate near the pulmonary veins. Due to impairments in the left atrial electrical substrate, reentrant circuits continuously activate the atrial myocardium much faster than what occurs during sinus rhythm. The vulnerability of the atrium to fibrillate in response to ectopic foci is influenced by factors including tissue architecture, such as size of the left atrium and presence of fibrosis, as well as intrinsic properties of the cardiomyocytes affecting impulse propagation (sodium current and gap junction expression) and action potential duration [18]. There are multiple scales for assessing the contributors that give rise to an arrhythmic substrate at a chamber-specific level, including single cell analysis, cardiac slices, cardiac wedges, and intact whole hearts. We later discuss the techniques used to measure regional heterogeneity in normal and diseased hearts, the advantages and disadvantages of each method, and discuss under which experimental circumstances each technique might be useful.

### 4.2. Isolated Cardiomyocytes

Regional EP heterogeneity can be measured by isolating cardiomyocytes from different chambers through tissue digestion with enzymes to break down the extracellular matrix and connective tissues, followed by micro-dissection. Using this technique, one can perform expression studies along with isolated patch clamp recording to characterize EP parameters, such as individual ionic currents and APs. Alterations to AP characteristics such as resting membrane potential, *dV_m_/dt_max_* (maximal upstroke velocity, or the slope of phase 0 of the cardiomyocyte AP mainly dictated by Na^+^ current), AP amplitude, and APD at multiple stages of repolarization, can provide insight into which ionic currents may be altered in an experimental model. There are several types of single cell patch clamp configurations one can use (whole cell, outside-out patch, perforated patch, etc.) depending on the experimental question. In-depth descriptions of EP principles and an overview of the single cell patch clamp methods are available, and are outside the scope of this review [19].

Recent studies have further probed EP heterogeneity by isolating cardiomyocytes from different layers of the chamber walls, therefore allowing characterization of EP properties of subepicardial, midmyocardial, and subendocardial myocytes separately [20,21]. It has been previously shown that these cardiomyocyte populations have distinct AP waveform morphologies and may, therefore, be differentially affected in disease states. For instance, BrS has been associated with dysregulation of the transcription factor *HEY2*, which affects the expression of subunits comprising the outward K^+^ current, I_to_ [20]. Using isolated single cells to separate the subepicardium and subendocardium of the RV in *Hey2*^+/−^ heterozygous knockout mice, Veerman et al. determined that loss of Hey2 differentially affects subepicardial cardiomyocytes, resulting in their EP properties becoming more like subendocardial cardiomyocytes [20]. Without separating the different parts of the RV wall, changes in the transmural EP gradient would not have been identified. Recent studies are applying similar techniques to understand how ionic currents are differentially regulated between cardiac chambers in normal and diseased states in humans. For example, Johnson and Springer et al. utilize this technique to demonstrate that the transient K^+^ current, I_to,fast_, which is normally differentially regulated across the chamber wall in non-failing human LV, is remodeled specifically in failing LV subepicardial cardiomyocytes leading to attenuation of the normal transmural gradient [21].

The advantages of using isolated cardiomyocytes include the ability to analyze cells from all layers of the cardiac wall within the same animal. Furthermore, one can isolate cells from regions of the heart anatomically difficult to access using tissue preparations, such as pacemaker cells from the sinoatrial node (SAN) or Purkinje cells within the ventricle. Single-cell isolation is the primary technique used to study specific ionic currents in the heart, however, the enzymatic digestion is harsh on cell viability and can alter the cellular expression profile and/or partially digest transmembrane proteins altering ionic currents. Another disadvantage is loss of endogenous physical and electrical coupling with nearby cells, which ultimately influences cellular EP properties. To overcome this disadvantage, a novel technique, known as loose patch photolysis, was recently developed by the Escobar laboratory that enables ionic currents and kinetics to be measured in intact tissues [22]. Sharp microelectrode recordings, detailed later in this review, also allow for single-cell EP measurements at the tissue and organ level.

### 4.3. Organotypic Cardiac Slices

Recently, there has been increasing interest in the use of organotypic slices as a model system for studying cardiac physiology. Unlike isolated cardiomyocytes, tissue architecture and electrical coupling with surrounding cells is maintained in cardiac slices, thereby allowing measurements of impulse propagation. Cardiac slices are typically between 100–400 μm, and given the diffusion limits of oxygen through tissue, slice viability can be maintained via superfusion, eliminating the need for vascular perfusion. Organotypic cardiac slices have been studied from small and large animals including mice, rats, guinea pigs, rabbits, dogs, and pigs, as well as from human heart biopsies and human hearts not suitable for transplantation [23,24,25]. Many organotypic slices can be obtained from a single heart from multiple locations within the LV and RV chamber walls, which allows regional-specific physiological studies from the same heart. Recent methodology for culturing cardiac slices while maintaining electrical viability has opened new avenues for therapeutic discovery and mechanistic testing in electrically mature human tissue, with potential applications including acute and chronic drug studies and gene therapy approaches [24]. While slices can be used to study cardiac conduction and APD, the tissue volume is not sufficient for studying arrhythmia mechanisms. In addition, donated human hearts unsuitable for transplantation are an extremely precious resource, and the unpredictable availability and infrastructure required for rapid procurement for subsequent physiologic studies are potential barriers for widespread adoption.

### 4.4. Cardiac Wedge Preparations

Similar to the cardiac slice preparation, wedge preparations preserve the native tissue architecture with the advantage of traversing the entire thickness of the chamber, thereby accommodating studies which include arrhythmia inducibility. Major drawbacks include the need for a coronary artery for cannulation and perfusion of oxygenated physiologic solution, thereby limiting its use on atrial preparations and its use on small animals. In addition, while cardiac slices can be cultured for multiple days, a cardiac wedge is viable for hours, thus imposing time limitations on experiments.

### 4.5. Intact Whole Hearts

Langendorff-perfused whole hearts can be used to study cardiac physiology and arrhythmia susceptibility, and in some instances, provide the most relevant ex vivo model. Sick Sinus Syndrome (SSS), otherwise known as SAN dysfunction, is characterized by a slow heart rate, sinus pauses, and susceptibility to supraventricular tachycardia [26]. Several genetic and pacing-induced models of SSS have been generated and characterized via patch clamp recordings performed on isolated SAN cells, and these experiments delineated alterations in ionic currents within the SAN cells responsible for the slow heart rate [27,28]. In contrast, a recent study by Qiao and Lipovsky et al. found that in a model of Notch-induced SSS, the SAN itself is not the main contributor to the disease phenotype [29]. In this case, decreased excitability of the atrial myocardium prevents the SAN “source” from efficiently activating the atrial “sink”. We performed a series of experiments including sharp microelectrode intracellular recordings on atrial myocardium of Langendorff-perfused hearts to demonstrate altered excitability, and programmed stimulation to demonstrate susceptibility to supraventricular tachycardia, while single-cell isolation of SAN cells would not have identified the primary alteration underlying SSS in this model.

### 4.6. Sharp Microelectrode Intracellular Recordings on Intact Cardiac Tissue

Like isolated cardiomyocyte recordings, sharp microelectrode intracellular recordings are used to probe the electrical activity at a single-cell level. However, sharp microelectrode recordings can be performed on intact tissue. The microelectrodes used are called “sharp” because they penetrate the cell membrane without significant damage to cellular function as the membrane quickly seals itself around the microelectrode. Sharp microelectrodes allow one to assess the EP characteristics of tissue slices, wedges, or intact hearts by impaling a single cell and measuring APs of that particular cell without subjecting the tissue to a digestion protocol. The AP characteristics mentioned previously that can be obtained when doing single cell patch clamping can also be measured using sharp microelectrodes on intact tissue. For example, as mentioned above, Qiao and Lipovsky et al. utilized sharp microelectrode intracellular recordings on Langendorff-perfused intact mouse hearts to demonstrate that the *dV_m_/dt_max_* of atrial cardiomyocytes is significantly slower after transient induction of Notch signaling, suggesting a functional impairment in Na^+^ current [29]. Notch induction reduces *Scn5a* expression in the right atrial myocardium, and since mutations within *Scn5a* have been linked to familial SSS, this is a plausible mechanism for heart rate slowing after transient Notch activation [30]. Addition of pharmacologic agents to the superfusate or perfusion solution of slices, wedges, or intact hearts followed by sharp microelectrode recordings can further probe how drugs affect AP morphology [31,32]. While significant information on AP characteristics can be measured using sharp microelectrodes, impulse propagation, and activation patterns of the heart are not visualized.

### 4.7. Optical Mapping

While recording EP parameters from single cells provides key insights, optical mapping is a technique that can be used to assay EP parameters in cardiac slices, wedge preparations, and whole-organ level physiology. Using voltage-sensitive dyes and high-speed cameras, optical APs can be measured with high spatial and temporal resolution without the need for recording electrodes [33]. In addition to the use of dyes to track membrane potential, calcium-handling dyes can also be utilized to provide nearly real-time imaging of calcium flux [24]. In Langendorff-perfused heart preparations, optical mapping can delineate baseline activation and repolarization patterns during sinus rhythm, providing information with respect to both time and space. Visualization of these patterns is achieved with an isochrone map which utilizes a color scale to represent activation times; therefore, regions of tissue activating at the same time are shown as the same color (isochrones). Further programmed stimulation of specific regions of the heart during the mapping protocol can interrogate parameters such as local conduction velocity (CV) and effective refractory periods (ERPs). Alterations in these parameters can predispose to arrhythmias, which can be further elicited via optical mapping using more aggressive stimulation protocols [29,34].

**Figure 1:**
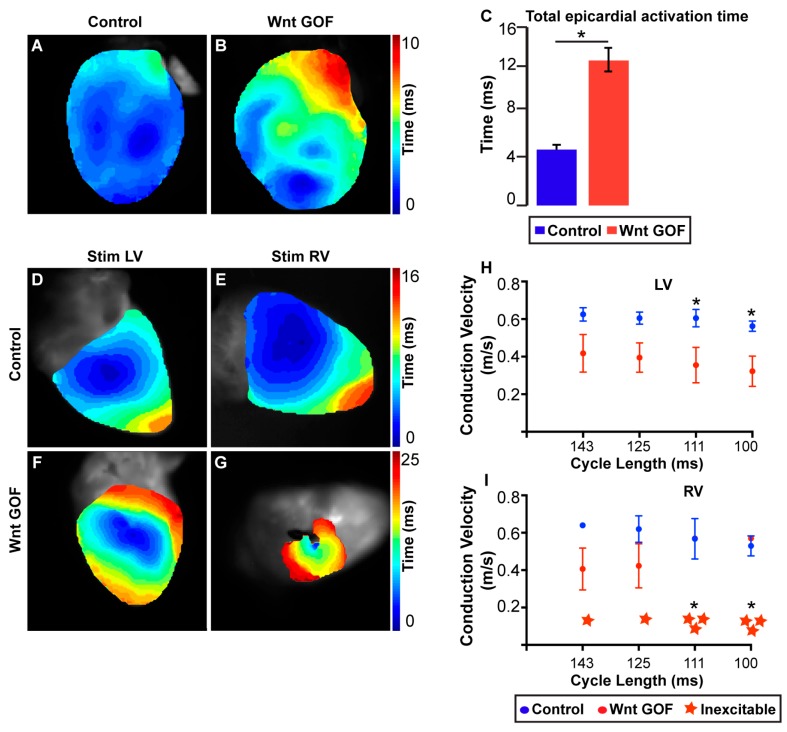
**Ectopic Wnt activation differentially affects the electrical phenotype of RV versus LV.** (**A**,**B**) Reconstructed electrical activation pattern from optical mapping experiment during sinus rhythm in control (**A**) and Wnt GOF (**B**) mice. (**C**) Total epicardial activation time is significantly prolonged in Wnt GOF mice (4.4 ± 0.4 versus 11.5 ± 1.0 ms, *n* = 4 each genotype). (**D**–**G**) Representative electrical activation pattern of the LV and RV during epicardial stimulation in control (**D**,**E**) and Wnt GOF (**F**,**G**) mice. (**H**) Left ventricular (LV) longitudinal conduction velocity of Wnt GOF mice was slower during stimulation at each cycle length, and the difference between the two genotypes became larger at faster pacing rates (111 and 100 ms cycle lengths, *n* = 4). (**I**) Right ventricular (RV) longitudinal conduction velocity of Wnt GOF mice was also slower, and was more severely decreased than in the LV. One Wnt GOF mutant had an electrically inexcitable RV when paced at 143 ms cycle length, while two others had markedly decreased conduction velocity at slower cycle lengths and became inexcitable at pacing rates above 125 ms cycle interval. This is consistent with decremental conduction, a property of AV canal and AV nodal tissues. Note the different time scales between each experiment. Data are represented as mean ± SEM. Group comparison for conduction velocity was performed using a Student’s unpaired 2-tailed *t*-test at each cycle length. Group comparison for inexcitability was performed using a Chi squared test without Yate’s correction. * *p* < 0.05. *Modified from Gillers et al., Canonical Wnt Signaling Regulates Atrioventricular Junction Programming and Electrophysiological Properties, Circulation Research, Volume 116, Issue 3, page 402. http://circres.ahajournals.org/cgi/pmidlookup?view=long&pmid=25599332*

Optical mapping of genetically mutant mouse hearts has been used extensively to study regional-specific conduction abnormalities [35]. For example, after observing an abnormal ECG pattern indicative of slowed ventricular activation in mice where the Wnt signaling pathway was activated during development, Gillers et al. used optical mapping to directly investigate the total epicardial activation time as well as CV within both ventricular chambers. While CV was decreased in both the LV and RV of Wnt activated mice, the RV was much more severely affected (Figure 1) [35]. Future experiments may provide more insight into mechanisms for how developmental programming influences these Wnt-mediated chamber-specific electrophysiologic effects.

In summary, optical mapping is a versatile technique that can be applied to the study of cardiac electrical activity at both the tissue and whole-organ level. The relative simplicity of the setup combined with the availability of open-source analysis software has increased the availability and favorability of this technique to laboratories outside of the traditional EP field. A main disadvantage of this technique is the inability to measure at multiple depths. Multi-photon microscopy has been recently used to measure electrical activity and calcium transients simultaneously at varying tissue depths in intact Langendorff-perfused hearts, allowing transmural EP differences to be studied with high spatial and temporal resolution [36,37]. Although both techniques can only be performed on explanted hearts, other techniques have been developed to allow measurement of cardiac electrical properties in vivo and are discussed below.

### 4.8. Optogenetics

Optical mapping utilizes cardiotoxic voltage-sensitive dyes which limits the ability to perform sequential experiments on a single sample ex vivo. Optogenetics combines the utility of optical electrophysiology with genetics and circumvents the use of cardiotoxic dyes, enabling electrophysiologic measurements in vivo through the use of genetically-encoded voltage indicators (GEVIs). GEVIs are proteins that can sense voltage changes and have been genetically engineered to express an indicator, such as fluorescence, in response to voltage changes. In addition to GEVIs, genetically encoded calcium indicators (GECIs), such as GCaMP2, have been developed and utilized to visualize Ca^2+^ transients and study conduction abnormalities in mice in vivo [38,39]. Optogenetics can also provide an alternative to electrical stimulation or inhibition of cardiomyocyte depolarization utilizing microbial opsins, called optogenetic actuators. Two such examples include channel rhodopsins used to generate depolarizing/excitatory currents, and chloride pump halorhodopsin which can generate a hyperpolarizing/inhibitory current. These actuators are single membrane-bound proteins that can function as ion channels in complex with a form of Vitamin A (retinal) when exposed to light [40,41]. This technique provides cell specificity and spatiotemporal precision in manipulating cardiomyocyte stimulation. In 2010, Bruegmann et al. optimized and validated this method to demonstrate optogenetic control of beating rate in embryonic stem cell-derived cardiomyocytes, as well as in transgenic cardiac tissue [42]. Recently, several studies have shown optogenetic defibrillation of ventricular tachycardia in mouse and rat models ex vivo, providing evidence for its potential use in clinical cardiology [43,44,45]. However, major challenges for clinical application include—(i) toxicity of the gene to be transferred; (ii) the requirement for endogenous retinal to form a functional complex; (iii) an effective gene delivery method; and (iv) delivery of a light source compatible for each individual heart.

### 4.9. Electrocardiographic Imaging (ECGI)

Electrocardiographic imaging (ECGI) is a non-invasive imaging procedure that can map electrical activity on the cardiac epicardial surface in humans. A multi-electrode vest records 250 body-surface electrocardiograms (ECGs), which can be combined with computed tomography (CT) or magnetic resonance imaging (MRI) to provide cardiac geometry. This technology can provide patient-specific activation maps and repolarization patterns of the epicardial surface and has been used to study hereditary arrhythmias such as Long QT syndrome (LQTS) and BrS, as well as acquired arrhythmias such as post-myocardial infarction ventricular tachycardia [46,47,48,49]. ECGI provides information on both cardiac activation and repolarization, somewhat similar to optical mapping.

**Figure 2:**
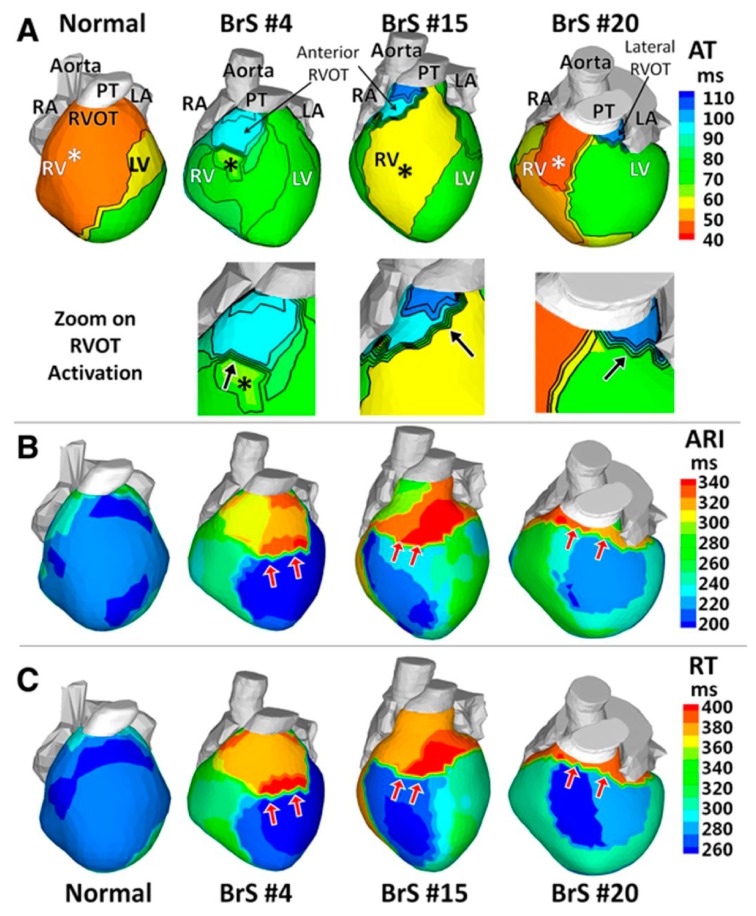
**Activation and repolarization during sinus rhythm.** (**A**) Activation times isochrone maps (AT). Insets, zoom on the RVOT. (**B**) Activation-recovery interval maps (ARI). (**C**) Recovery time (RT) maps. Epicardial breakthroughs are indicated by asterisks. Isochrones are depicted in thin black lines. Black arrows in the RVOT zoom maps of **A** point to slow conduction indicated by crowded isochronal lines. Red arrows in **B** and **C** point to regions with steep repolarization gradients. BrS indicates Brugada syndrome; LA, left atrium; LV, left ventricle; PT, pulmonary trunk; RA, right atrium; RV, right ventricle; and RVOT, right ventricular outflow tract. *Modified from Zhang et al., Cardiac electrophysiological substrate underlying the ECG phenotype and electrogram abnormalities in Brugada syndrome patients, Circulation, Volume 131, Issue 22, page 1954. http://circ.ahajournals.org/content/131/22/1950.long*

Zhang et al. used ECGI to delineate the cardiac electrophysiological substrate underlying the BrS phenotype [48]. BrS patients exhibit delayed right ventricular outflow tract (RVOT) activation and slow conduction, prolonged RVOT recovery time and activation-recovery intervals indicating delayed repolarization, as well as steep repolarization gradients at RVOT borders (Figure 2). This study further supports the concept that regional-specific changes in electrophysiologic substrate within the RVOT underlie BrS. In addition to aiding our understanding of underlying mechanisms of arrhythmias, ECGI has recently been used to non-invasively map ventricular tachycardia prior to delivery of radiation for tachycardia ablation [50]. Given the wealth of information that can be gleaned non-invasively, we expect ECGI will gain more widespread use in both research and clinical applications tailored to personalized therapies.

## 5. Conclusions

In summary, the evolution of technology and experimentation has revealed a greater appreciation of regional heterogeneity in the heart during health and disease. To fully understand the basis for regulation of cardiac electrophysiology, it is imperative to integrate concepts and techniques from diverse disciplines, including clinical cardiology, biomedical engineering, physiology, genomics, and developmental biology. Filling these gaps in our knowledge is challenging and requires us to understand several languages, but is nonetheless crucial to maximizing our collective understanding and to prevent potential new insights from being “lost in translation”.
